# Spatiotemporal gait compensations following medial collateral ligament and medial meniscus injury in the rat: correlating gait patterns to joint damage

**DOI:** 10.1186/s13075-015-0791-2

**Published:** 2015-10-14

**Authors:** Heidi E. Kloefkorn, Brittany Y. Jacobs, Ayomiposi M. Loye, Kyle D. Allen

**Affiliations:** J. Crayton Pruitt Family Department of Biomedical Engineering, University of Florida, 1275 Center Drive, Biomedical Sciences Building JG56, Gainesville, FL 32610 USA; Institute of Cellular Engineering and Regenerative Medicine, University of Florida, Gainesville, FL USA; Nanoscience Institute for Medical and Engineering Technology, University of Florida, Gainesville, FL USA; Pain Research and Intervention Center of Excellence, University of Florida, Gainesville, FL USA

## Abstract

**Introduction:**

After transection of the medial collateral ligament and medial meniscus (MCLT + MMT) in the rat, focal cartilage lesions develop over 4–6 weeks; however, sham surgery (MCLT alone) does not result in cartilage damage over a similar period. Thus, comparison of MCLT + MMT with the MCLT sham group offers an opportunity to investigate behavioral modifications related to focal cartilage and meniscus damage in the rat.

**Methods:**

MCLT or MCLT + MMT surgery was performed in the right knees of male Lewis rats, with spatiotemporal gait patterns and hind limb sensitivity assessed at 1, 2, 4, and 6 weeks postsurgery (n = 8 rats per group per time point, n = 64 total). After the animals were euthanized, Histology was performed to assess joint damage.

**Results:**

MCLT + MMT animals had unilateral gait compensations at early time points, but by week 6 bilateral gait compensations had developed in both the MCLT sham and MCLT + MMT groups. Conversely, heightened tactile sensitivity was detected in both MCLT sham and MCLT + MMT animals at week 1, but only the MCLT + MMT animals maintained heightened sensitivity to week 6. Cartilage lesions were found in the MCLT + MMT group but not in the MCLT sham group. Correlations could be identified between joint damage and gait changes in MCLT + MMT animals; however, the same gait changes were found with MCLT sham animals despite a lack of joint damage.

**Conclusions:**

Combined, our data highlight a common conundrum in osteoarthritis (OA) research: Some behavioral changes correlate to cartilage damage in the OA group, but the same changes can be identified in non-OA controls. Of the behavioral changes detected, allodynia was maintained in MCLT + MMT animals but not in the MCLT sham group. However, the correlation between cartilage damage and hind limb sensitivity is relatively weak (*R* = −0.4498), and the range of sensitivity measures overlaps between groups. The factors driving gait abnormalities in MCLT and MCLT + MMT animals also remain uncertain. The gait modifications are similar between groups and do not appear until weeks after surgery, despite cartilage damage being focused in the MCLT + MMT group. Combined, our data highlight the need to evaluate the links between noncartilage changes and behavioral changes following joint injury in the rat.

**Electronic supplementary material:**

The online version of this article (doi:10.1186/s13075-015-0791-2) contains supplementary material, which is available to authorized users.

## Introduction

Because ligament and meniscus injuries significantly increase the risk for developing osteoarthritis (OA) [1, 2], surgical simulation of a ligament and/or meniscus injury is commonly used to model OA development in rodents [3–5]. In the rat knee, focal cartilage lesions form in the medial compartment several weeks after a surgically simulated radial tear of the medial meniscus or rupture of the anterior cruciate ligament [3, 4, 6]. Using histology, detailed assessment of joint remodeling is possible in these rodent OA models [7]; however, quantifying the symptomatic and functional consequences of joint injury can be difficult in rodent OA models.

Following a combined surgical transection of the medial collateral ligament (MCLT) and radial transection of the medial meniscus (MMT) in rats, fibrillation of the articular surface is seen at 1–2 weeks postsurgery, followed by the development of full-thickness cartilage lesions at 2–6 weeks postsurgery. Similarly, osteophytes form along the medial margin of the joint at 4–6 weeks postsurgery [3, 6, 8]. However, rats that received a sham surgery (MCLT alone) did not show evidence of cartilage loss or osteophyte formation over a similar time frame [3, 6]. Our group has previously described gait abnormalities in rats receiving MCLT + MMT surgery, wherein rats receiving MCLT + MMT surgery spent unequal time on their hind limbs (imbalanced stance time) and used asymmetric foot strike sequences after surgery (limping) [8]. Similar compensations were identified in animals in the MCLT sham surgery group; however, the magnitude of the compensation tended to vary between groups. As such, comparison of MCLT with MCLT + MMT in the rat may provide an opportunity to investigate a joint trauma that results in cartilage damage (MCLT + MMT) relative to a joint trauma that does not result in cartilage damage (MCLT sham), as well as how the specific effects of cartilage loss alter an animal’s behavior and gait.

Owing to the longitudinal design of our past experiment [8], histological grades were available only at the final time point, and, as such, correlative relationships between the detected gait abnormalities and osteoarthritic changes within the joint could not be constructed. In the present study, the correlative relationships between gait compensations and osteoarthritic remodeling within the joint were investigated in rats with MCLT alone and rats with MCLT + MMT. First, we provide a detailed characterization of the spatiotemporal gait pattern and tactile sensitivity for rats receiving MCLT sham or MCLT + MMT surgery at 1, 2, 4, and 6 weeks after surgery. Following this characterization of rodent gait, osteoarthritic remodeling within the joint is described, using the quantitative Osteoarthritis Research Society International (OARSI) histological grading system for the rat [7]. Finally, correlative relationships between gait compensations and joint remodeling in the MCLT sham and MCLT + MMT groups are investigated. Our data demonstrate that, whereas correlations exist between histological scores of joint damage and behavioral changes in the MCLT + MMT group, similar behavioral changes could be found without significant cartilage damage or osteophyte growth in the MCLT sham group. Combined, these data indicate cartilage damage and behavioral changes in the rodent may be coincidental in the MCLT + MMT model of OA and suggest that noncartilage mechanisms may be involved in the development of gait compensations following joint injury in the rat.

## Methods

### Experimental design and animal surgery

A total of 64 male Lewis rats (approximately 250 g) were obtained from Charles River Laboratories (Wilmington, MA, USA) and acclimated to the University of Florida housing facilities for 1 week before investigation. After acclimation, 32 rats received MCLT + MMT surgery and 32 rats received MCLT alone (MCLT sham), as previously described [3, 8]. The surgeries were performed in four 16-animal cohorts, with 8 MCLT + MMT surgeries and 8 MCLT sham surgeries performed for each surgical day. Briefly, animals were anesthetized in a 4 % isoflurane sleep box, prepared for aseptic surgery, and transferred to a sterile field with anesthesia maintained by mask inhalation of 2 % isoflurane. A medial midline skin incision was made on the right hind limb, and the medial collateral ligament (MCL) was exposed via blunt dissection and transected. At this point, the wounds of animals receiving MCLT alone (sham) were closed as described below. For animals receiving MCLT + MMT, the joint was placed in a valgus orientation to expose the central portion of the medial meniscus; then, a complete radial transection was performed in the central portion of the medial meniscus using a number 11 blade scalpel. For animals tested at 2, 4, and 6 weeks after surgery, the surgical site was closed with 9-mm wound clips, which were removed 10–14 days after surgery and thus were not present during behavioral testing. For animals tested at 1 week, 5-0 VICRYL sutures (Ethicon, Somerville, NJ, USA) were used to close the incision site. These sutures did remain in place during behavioral testing at 1 week. Spatiotemporal gait pattern and mechanical sensitivity testing was performed in eight MCLT sham and eight MCLT + MMT animals at 1, 2, 4, and 6 weeks after surgery, as described below (n = 8 per treatment per time point). The protocol for the surgical cohorts was carried out in the following order: week 4, week 1, week 2, and week 6. After behavioral testing, animals were euthanized by exsanguination while under deep anesthesia with knee joints collected for histological grading. All methods used in the study were approved by the University of Florida Institutional Animal Care and Use Committee and in conformance with Association for Assessment and Accreditation of Laboratory Animal Care recommendations on animal research.

### Spatiotemporal gait testing

**Fig. 1:**
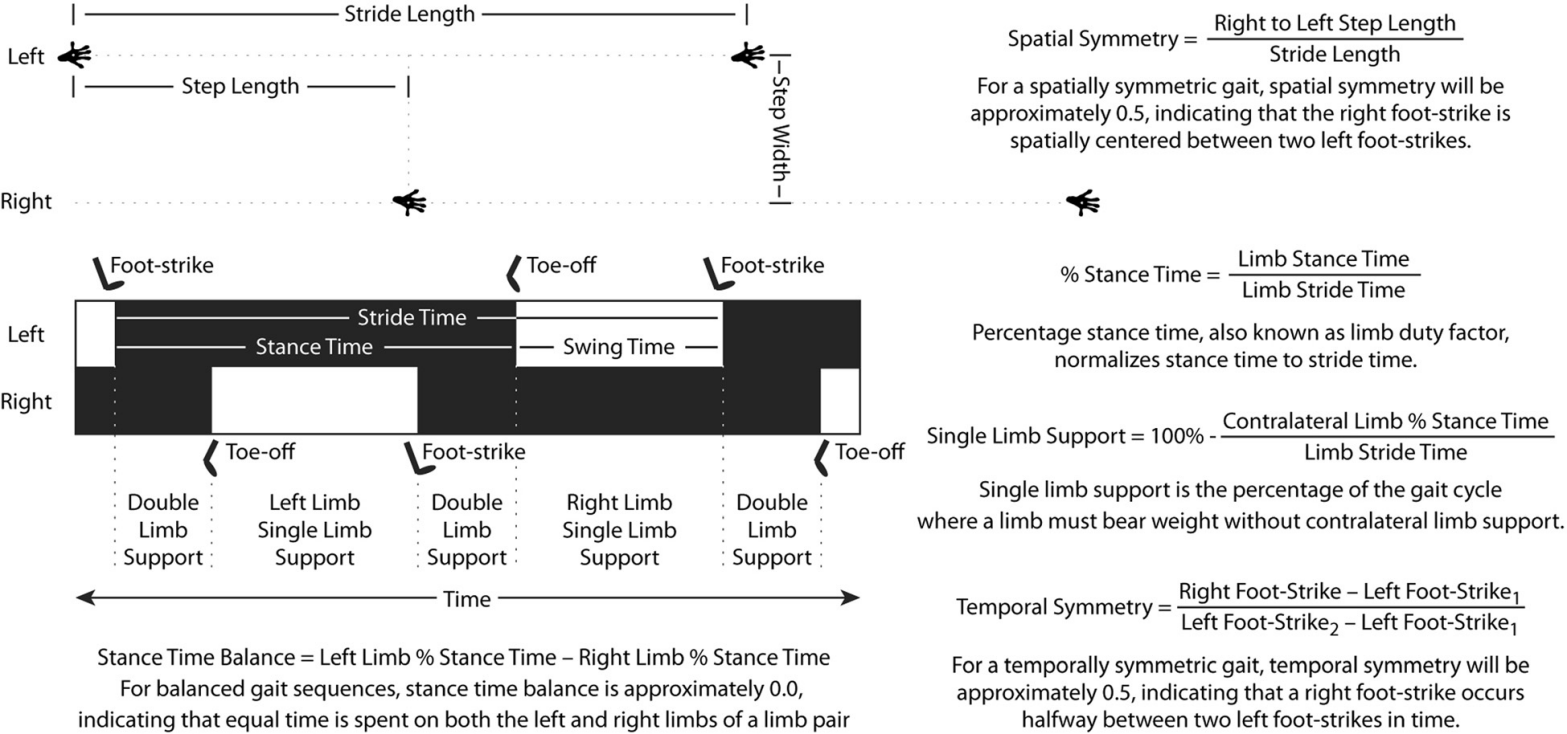
Spatiotemporal characteristics of gait sequence of a rat. Hind limb footprint pattern and temporal gait sequence of a rat is shown. The forelimb footprints and temporal gait sequence have been omitted for clarity. Common spatial characteristics of the rat include stride length, step width, and step length; however, because stride length and step length are strongly associated, spatial symmetry can be used to describe the placement of the right footprint relative to two left footprints. The temporal characteristics of a single limb include stride time, stance time, and swing time. Stance time is typically normalized to stride time, as percentage stance time (also known as *duty factor*) follows a more linear relationship to an animal’s walking velocity. Similarly, the single-limb support phase for a given limb is frequently assessed as relative to the gait cycle. The synchronicity of the gait cycle can be evaluated through stance time balance and temporal symmetry. The gait cycle is balanced when an animal spends equal time on its left or right limb, represented by a stance time balance of 0. A gait cycle is temporally symmetric when the right foot strike occurs halfway in time between two left foot strikes, represented by a temporal symmetry of 0.5

Animals were acclimated to the gait arena over a period of 3 days before the start of the experiment. Spatiotemporal gait parameters were then measured in all animals at their respective time points, as previously described and reviewed [8–11]. Briefly, animals were placed in a 60 × 18-in. open gait arena and allowed to freely explore without an external stimulus or food enticement. As animals voluntarily explored the gait arena, five videos of rodents walking were acquired with a high-speed camera (M3, 250 frames/s; RedLake, San Diego, CA, USA). A mirror set at a 45-degree angle under the transparent arena floor allowed for simultaneous recording from the side and beneath the animal. Videos that included a minimum of four complete gait cycles across a consistent walking speed were digitized by hand using the DLTdataviewer subroutine in MATLAB (MathWorks, Natick, MA, USA) [12]. Digitizers were blinded to the treatment groups during processing. Digitized data were processed to calculate the median value of the following parameters for each trial: velocity, stance time, swing time, stride time, stride length, and step width. Using these parameters, spatial symmetry, temporal symmetry, percentage stance time, stance time balance, and the percentage of the gait cycle dedicated to single-limb support were calculated as previously reviewed [11] and described in Fig. 1. On the basis of past data demonstrating limited changes to the forelimb spatiotemporal characteristics of rodents due to a hind limb injury [8–11, 13–16], this work focuses on spatiotemporal changes in the hind limbs only.

**Fig. 2:**
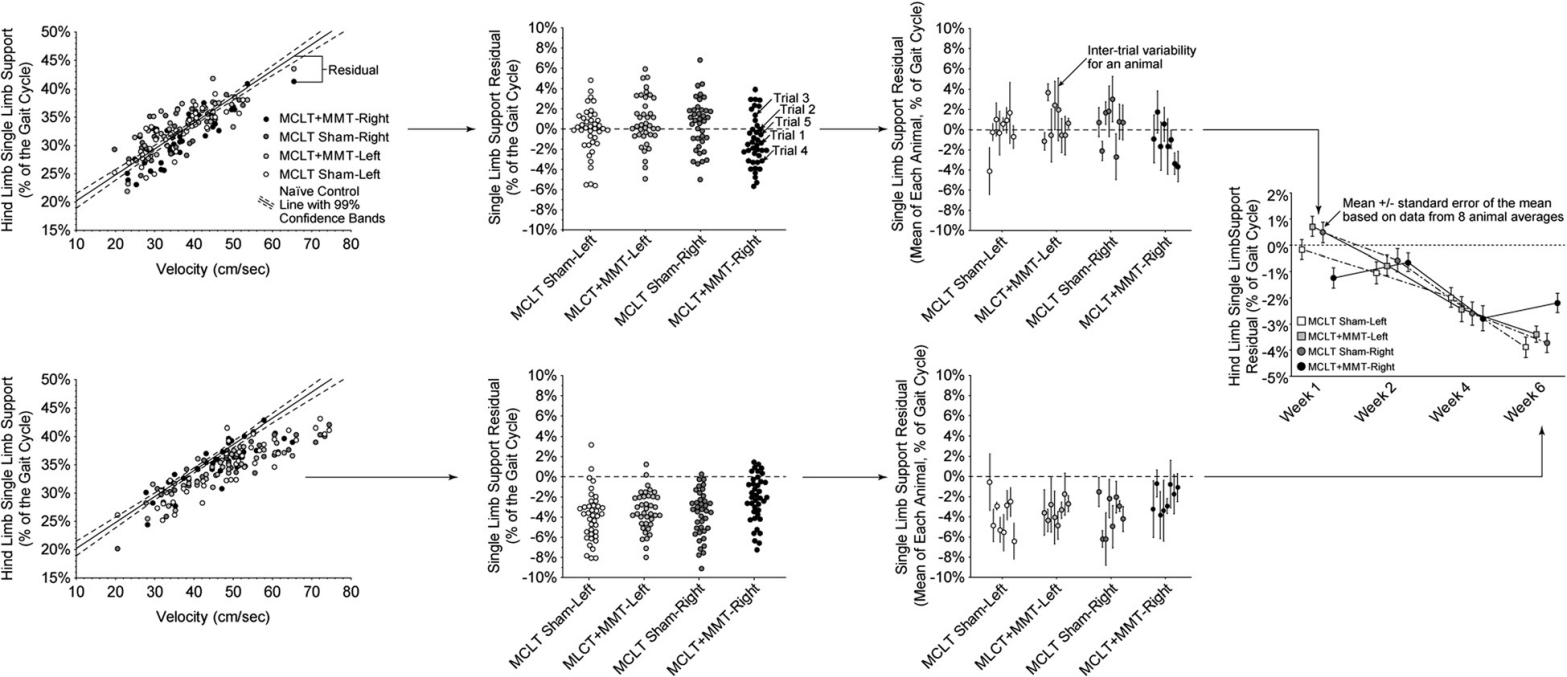
Conversion of velocity- and weight-dependent gait parameters into velocity- and weight-independent residuals. Stride length, step width, percentage stance time, and the single-limb support phase are known to strongly correlate with an animal’s walking velocity and size. Failure to account for the effects of velocity and weight in the analysis of a rodent’s gait will reduce the sensitivity of subsequent statistical analyses owing to an increase in unexplained variance (or mean squared error) [11]. To account for the effects of animal size and walking velocity, stride length, step width, percentage stance time, and the single-limb support phase were normalized to the predicted gait profile of velocity- and weight-matched naïve Lewis rats. This normalization process is shown for single-limb percentage stance time at 1 week and 6 weeks postsurgery. The control line is based upon historical data for the gait characteristics of naïve Lewis rats (*solid line* with 99 % confidence bands). This database is available for download at bme.ufl.edu/labs/allen and represents 280 gait trials collected in 28 different naïve Lewis rats at 49 different weights over a period of 8 years. The control line is used to predict the stride length, step width, percentage stance time, and the single-limb support phase for a size- and velocity-matched Lewis rat. With this prediction, velocity- and weight-independent residuals of stride length, step width, percentage stance time, and the single-limb support phase can be calculated by subtracting the predicted value for a velocity- and weight-matched Lewis rat. Once data are transformed into velocity- and weight-independent residuals, multiple trials of a rat are averaged such that the average gait profile for an animal directly corresponds to single histological profile in the correlation analyses. These eight values for each group time point were used to construct the data presented in Figs. 3 and 5. *MCLT* medial collateral ligament transection, *MMT* medial meniscus transection

Stride length, step width, percentage stance time, and the single-limb support phase are known to be strongly correlated to an animal’s walking velocity and weight, and failure to account for these sources of variation can reduce the sensitivity of subsequent statistical analyses [11]. To account for the effects of animal size and walking velocity, stride length, step width, percentage stance time, and the single-limb support phase were normalized to the predicted gait profile of weight- and velocity-matched naïve Lewis rats. This normalization process is visually described in Fig. 2 using the percentage of the gait cycle dedicated to single-limb support. Our database on the gait characteristics of naïve Lewis rats is available for download at bme.ufl.edu/labs/allen and represents 280 gait trials collected in 28 different naïve Lewis rats at 49 different weights over a period of 8 years. In brief, gait trials collected on naïve Lewis rats were used to predict the stride length, step width, percentage stance time, and the single-limb support phase for a weight- and velocity-matched Lewis rat. With this prediction, velocity- and weight-independent residuals of stride length, step width, percentage stance time, and the single-limb support phase can be calculated by subtracting the predicted value for a weight- and velocity-matched Lewis rat from the measured value for a Lewis rat with MCLT sham or MCLT + MMT surgery. Once data were transformed into velocity- and weight-independent parameters, data for each rat were averaged such that the average gait profile for an animal directly corresponded to a single histological profile in the correlation analyses described below. To be clear, the leftmost graphs in Fig. 2 visually describe the correlation between velocity and single-limb support only; however, residual data in the remainder of Fig. 2 and the figures presented in the Results section are normalized to both weight and velocity.

### Mechanical sensitivity

Mechanical sensitivity was assessed using Chaplan’s up–down protocol for von Frey filaments in the experimental groups listed above and in eight naïve Lewis rat littermates (control) [17]. The littermate-matched naïve control rats used for the von Frey analysis were also included in the naïve rat gait database; however, because these animals were not able to fully replicate the weights and velocities observed in our study, the full database was used for the gait analysis. These naïve rats were eventually used in a separate experiment unrelated to the present study.

During von Frey testing, experimenters were blinded to the animal surgical group during the test. Briefly, animals were acclimated to a wire mesh–floored cage for 15 minutes before the application of the 4.0-g von Frey filament to the plantar region of each hindfoot. Using a von Frey filament series (0.6, 1.4, 2, 4, 6, 8, 15, and 26 g), a withdrawal tolerance sequence was constructed wherein a less stiff filament was applied following a paw withdrawal and a more stiff filament was applied following filament tolerance. Using these data, the force where withdrawal and tolerance are equally likely can be approximated through Chaplan’s approximation (50 % paw withdrawal threshold) [17].

### Histology

Following behavioral testing, animals were euthanized while under deep anesthesia. Operated and contralateral knees were dissected, fixed in 10 % neutral buffered formalin for 48 h at room temperature, decalcified in Cal-Ex reagent (Fisher Scientific, Pittsburgh, PA, USA) for 3 weeks at 4 °C, and then embedded in paraffin wax using vacuum infiltration. Sequential frontal sections (10 μm) were acquired on a rotary microtome, taking at least one section every 100 μm through the central regions of the knee. The central region was defined as sections past the anterior horn of the medial meniscus through to the posterior horn of the meniscus. Toluidine blue staining was conducted on central sections, with the section that represented the most severe degeneration on the tibial plateau selected for grading. Sections were graded using the OARSI histopathology scheme for the rat [7]. When called for by the OARSI histopathology scheme for the rat, pixels were converted to geometric distances using a calibrated digital recital. This scoring system is used to evaluate cartilage damage, osteophyte formation, synovial membrane inflammation, and growth plate changes through semiquantitative grading relative to sample images [18] and by measuring physical changes in cartilage and bone in the 2-D histological images [7].

### Statistical analysis

Dunnett’s test was first used to compare gait parameters with expected values or tactile sensitivity with preoperative controls, correcting for compounding type I errors caused by 8 or 16 comparisons with controls. Differences between surgical groups and across time were investigated using two-way analysis of variance test, followed by Tukey’s honestly significant difference post hoc test when indicated. Correlative relationships between histological measures and behavioral measures were constructed for MCLT sham and MCLT + MMT animals using univariate linear models. In order to obtain data that spans the range of histological damage and behavioral changes, correlation models were constructed across time points for both MCLT sham and MCLT + MMT animals (n = 32).

## Results

### Gait patterns

**Fig. 3:**
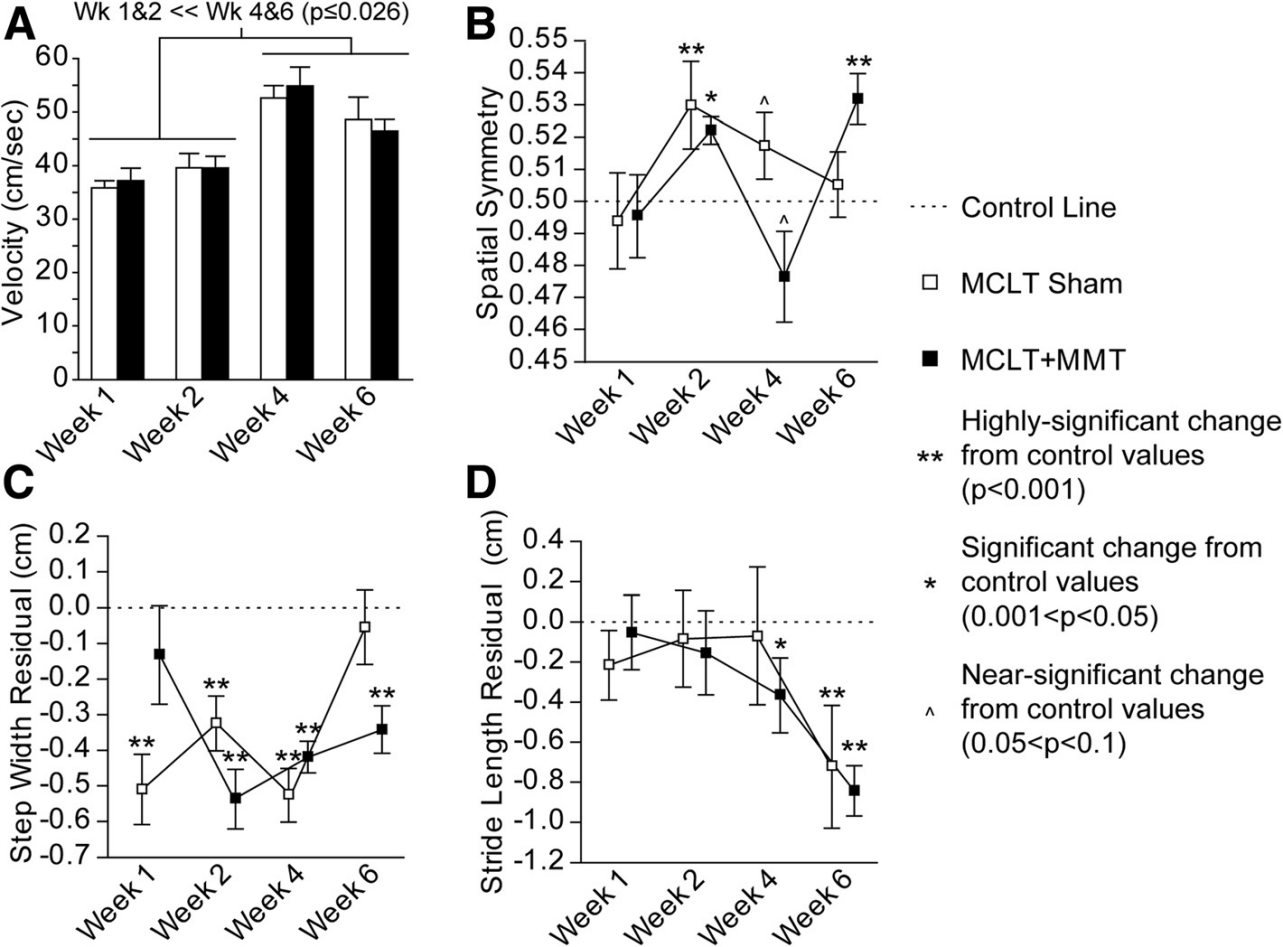
Spatial gait pattern changes following MCLT sham and MCLT + MMT surgery in the rat. Both MCLT sham and MCLT + MMT surgery resulted in altered spatial gait parameters in rats. **a** Velocity was significantly faster at week 4 and 6 relative to weeks 1 and 2 (*p* < 0.026). **b** Rats with MCLT + MMT surgery used spatially asymmetric foot strike patterns at week 2 and week 6, where the right step length was longer than expected (spatial symmetry >0.5; *p* = 0.005 and *p* < 0.001, respectively). At week 4, the spatial pattern of the MCLT + MMT group tended to be asymmetric, where the right step length was shorter than expected (*p* = 0.06). Spatial asymmetry indicative of longer right-step lengths were also seen in the MCLT sham at week 2 (*p* = 0.01) and tended to occur at week 4 (*p* = 0.06). **c** Step widths were narrower than expected in both groups, as indicated by a step width residual less than 0.0. The MCLT sham rats used narrower step widths at weeks 1, 2, and 4 (*p* < 0.001), but not at week 6. The MCLT + MMT animals used narrower step widths at weeks 2, 4, and 6 but not at week 1 (*p* < 0.001). **d** Stride lengths were shorter than expected in the rats with MCLT + MMT at 4 and 6 weeks (*p* = 0.04 and *p* < 0.001, respectively) and in the MCLT sham group at 6 weeks (*p* < 0.001), as indicated by a stride length residual less than 0.0. No significant differences were identified between the MCLT sham and MCLT + MMT groups within a specific time point. Data are presented as mean ± SEM. *MCLT* medial collateral ligament transection, *MMT* medial meniscus transection

**Fig. 4:**
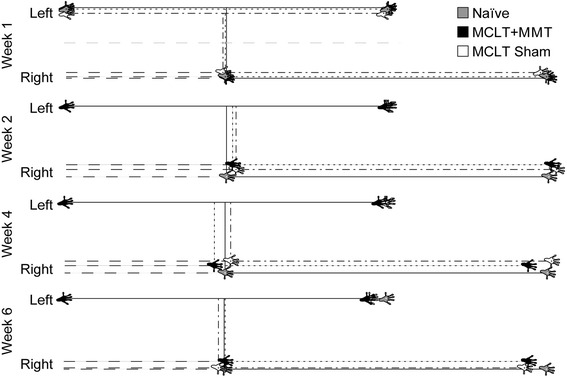
Visual representation of changes in the spatial gait pattern caused by MCLT sham and MCLT + MMT surgery in the rat. Using data for a naïve rat walking at an average velocity as a representative spatial pattern, shifts in the spatial pattern caused by MCLT sham or MCLT + MMT surgery are plotted for a rat of the same weight walking at the same velocity. At week 1, the patterns are similar between groups. At week 2, step widths begin to narrow in both MCLT sham and MCLT + MMT animals. At week 4, stride length begins to shorten in the MCLT + MMT group (compare second left footprint between groups). At week 6, stride lengths are significantly reduced in both the MCLT sham and MCLT + MMT groups (compare second left footprint between groups). Unfortunately, owing to the left-justified nature of this plot, spatial asymmetries (left-to-right step length divided by stride length) are difficult to visualize because of the concurrent changes in stride length. *MCLT* medial collateral ligament transection, *MMT* medial meniscus transection

Spatiotemporal gait abnormalities were detected in both rats that underwent MCLT sham surgery and rats with MCLT + MMT. Spatial compensations are graphically summarized in Fig. 3 and visually summarized in Fig. 4. Whereas walking velocity varied between time points, no velocity differences were found between MCLT sham and MCLT + MMT surgery within a given time point (Fig. 3a); however, the velocity differences between time points and the strong correlation between most gait parameters and velocity highlight the importance of standardizing spatiotemporal gait parameters to the animal’s selected velocity (see Fig. 2).

Spatial symmetry is used to investigate the geometric symmetry of the foot strike pattern, where a value near 0.5 indicates a right hind limb footprint is approximately halfway between two left hind limb footprints along the direction of travel (see Fig. 1). Whereas gaits were spatially symmetric at 1 week postsurgery, animals with MCLT + MMT surgery were spatially asymmetric at 2 and 6 weeks postsurgery (*p* = 0.005 and *p* < 0.001, respectively) and tended to be spatially asymmetric at 4 weeks postsurgery (*p* = 0.06) (Fig. 3b). MCLT sham surgery caused spatially asymmetric gaits at 2 weeks postsurgery (*p* = 0.01), and animals in this group tended to be spatially asymmetric at 4 weeks postsurgery (*p* = 0.10) but not at 6 weeks postsurgery.

Step widths were narrower than expected in the MCLT sham animals at 1, 2, and 4 weeks postsurgery (*p* < 0.001) but not at 6 weeks postsurgery. MCLT + MMT resulted in step widths that were comparable to historical controls at 1 week postsurgery but were narrower at 2, 4, and 6 weeks postsurgery (*p* < 0.001) (Fig. 3c). Narrower hind limb step widths may indicate that animals are using their forelimbs primarily for balance. Stride length residuals were reduced with MCLT + MMT at 4 and 6 weeks (*p* = 0.04 and *p* < 0.001, respectively) and with MCLT sham at 6 weeks (*p* < 0.001) (Fig. 3d).

**Fig. 5:**
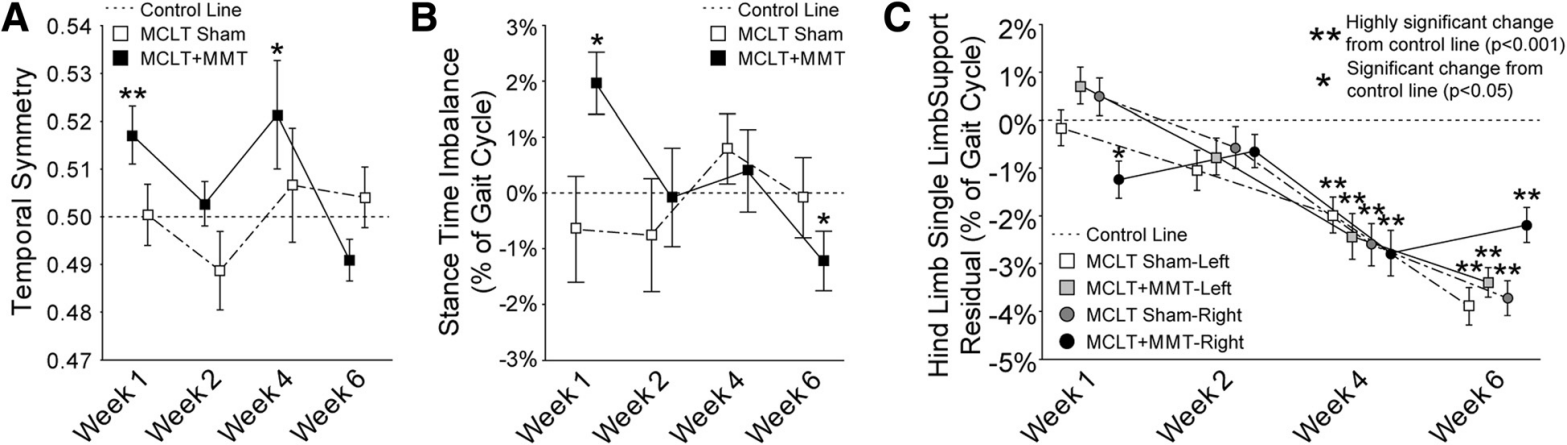
Temporal gait pattern changes following MCLT sham and MCLT + MMT surgery in the rat. **a** Temporal symmetry was used to investigate the synchronicity of the foot strike pattern in time, where a temporal symmetry near 0.5 indicates the foot strike of the right limb temporally occurs halfway between two left limb foot strikes. At week 1 and week 4, temporal asymmetries were greater than 0.5 in the MCLT + MMT group (*p* < 0.001 and *p* = 0.03, respectively), indicating that the time to transition from left to right foot strike was longer than the time to transition from right to left foot strike. **b** Stance time imbalance occurs when more time is spent on one limb relative to its contralateral limb. An imbalance greater than 0.0 was observed at week 1 in the MCLT + MMT group (*p* = 0.003), indicating that more time was spent on the left limb than on the right limb. Conversely, an imbalance less than 0.0 was observed at week 6 in the MCLT + MMT group (*p* = 0.048), indicating that more time was spent on the right limb than on the left limb. **c** Temporal gait compensations generally reduce the single-limb support phase spent on the injured limb. In the case of unilateral injuries, a reduced single-limb support phase is observed on the injured limb only. In conjunction with stance time imbalance findings in **b**, reduced right, but not left, single-limb support phases were found in the MCLT + MMT group at week 1 (*p* = 0.03). In bilateral compensations, single-limb support is reduced on both limbs of a limb pair. A bilateral compensation can be observed through reduced single-limb support phases in both limbs of both the MCLT sham and MCLT + MMT groups at week 4 and week 6 (*p* < 0.001 in all groups). Data are presented as mean ± SEM. *MCLT* medial collateral ligament transection, *MMT* medial meniscus transection

**Fig. 6:**
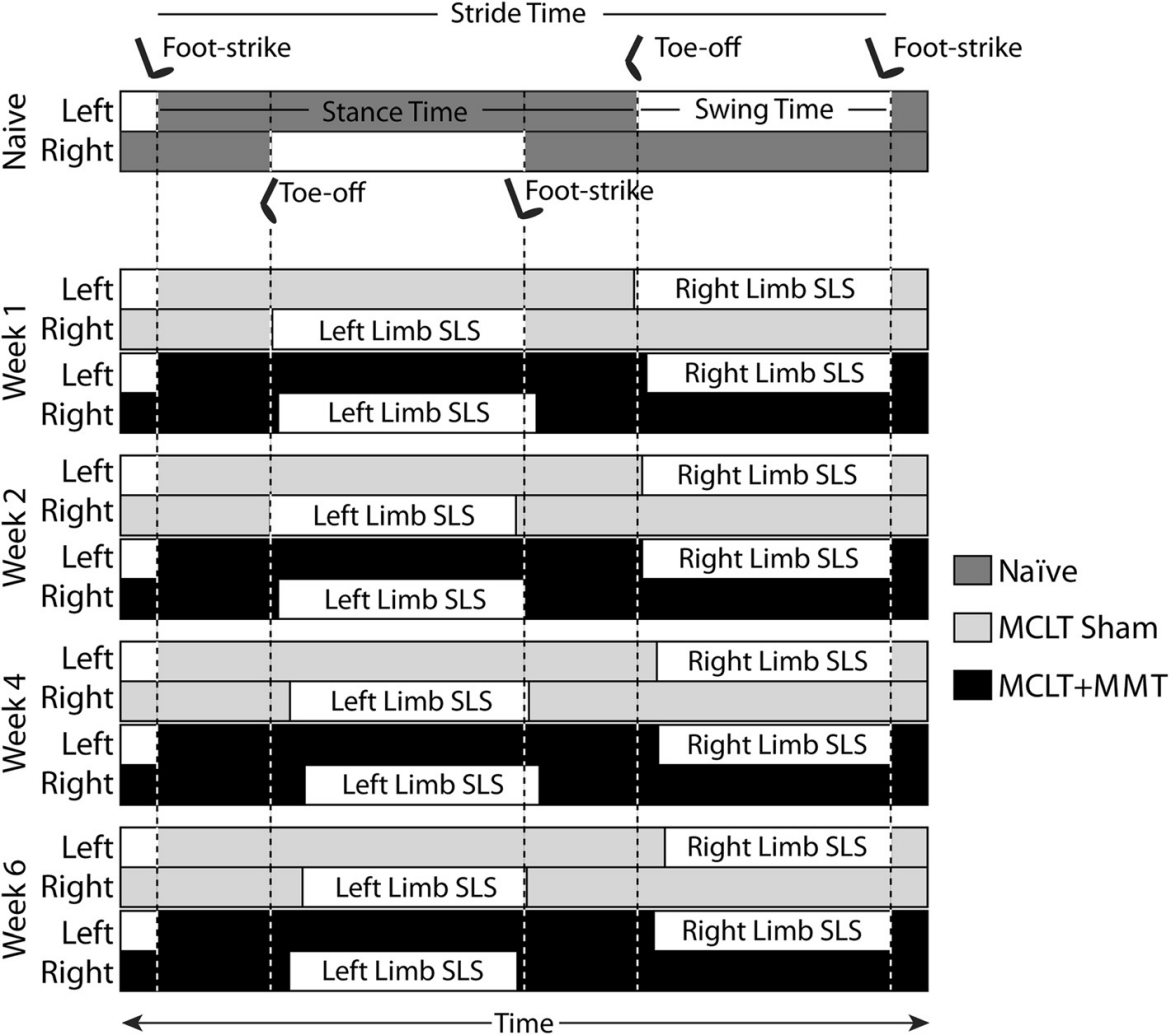
Visual representation of changes in the temporal gait pattern caused by MCLT sham and MCLT + MMT surgery in the rat. As in Fig. 4, data for an average-size naïve rat walking at an average velocity were used to construct a representative hind limb foot strike sequence for a walking rat using a modified Hildebrand chart (*top*). In this chart, the left hind limb foot strikes occur at the same time in each group. Using this presentation, temporal asymmetries can be observed by the shift of the right hind limb foot strike later in time for the MCLT + MMT group at week 1 and week 4. Because the right hind limb single-limb support phase (Right Limb SLS) occurs during the swing phase of the left hind limb, reduced right hind limb single-limb support can be observed via a delayed toe-off in the left hind limb in the MCLT + MMT group at week 1 and in both MCLT sham and MCLT + MMT groups at week 4 and week 6. Conversely, left hind limb single-limb support (Left Limb SLS) occurs during the swing phase of the right hind limb. As with spatial symmetry in Fig. 4, alterations in left hind limb single-limb support are more difficult to see, owing to the left foot strike standardization. *MCLT* medial collateral ligament transection, *MMT* medial meniscus transection

Temporal changes are graphically summarized in Fig. 5 and visually summarized in Fig. 6. Stance time imbalance and single-limb support residuals are presented as a percentage of the gait cycle, and this should not be confused with percentage change. For example, a single-limb support phase for a hind limb at walking velocities must be between 1 % and 50 % of the gait cycle; limb swing times over 50 % of the gait cycle are defined as running gaits. On the basis of historical data collected for naïve Lewis rats, the range of single-limb support is 18.7–50 % of the gait cycle. Thus, a 2 % imbalance or 3 % single-limb support residual in expected single-limb support is a significant magnitude relative to the range of possible values for temporal gait parameters (approximately 32 % of a gait cycle).

As with spatial symmetry, temporal symmetry investigates the symmetry of the foot strike pattern in time, where a temporal symmetry value near 0.5 indicates an animal’s right foot strike occurs approximately halfway between two left limb foot strikes in time (see Fig. 1). Temporal asymmetries were detected in the MCLT + MMT group at week 1 and week 4 (*p* < 0.001 and *p* = 0.03, respectively) (Fig. 5a). Similarly, stance time imbalance occurs when an animal spends more time on one limb relative to the contralateral limb (see Fig. 1). In our animals, stance time imbalance was observed in the MCLT + MMT group at week 1 and week 6 (*p* = 0.003 and *p* = 0.048, respectively) (Fig. 5b).

### Evidence of unilateral and bilateral gait compensations

Unilateral compensations occur when the contralateral limb compensates for an injured limb. Conceptually, this is similar to a limp associated with a sprain or muscle strain. Unilateral compensations are identified primarily by temporal asymmetry and stance time imbalance and to a lesser degree by spatially asymmetric footprint patterns [11]. The strongest evidence of unilateral compensation was seen in the MCLT + MMT group at week 1, including a combined temporal asymmetry and stance time imbalance. At week 2, both MCLT sham and MCLT + MMT animals used spatially asymmetric patterns that were temporally symmetric and balanced, which is indicative of altered foot placement but not limb disuse. At week 4, MCLT sham and MCLT + MMT animals used balanced gaits, with a tendency toward spatially asymmetric foot placement. MCLT + MMT animals also had temporally asymmetric gait patterns. Overall, this pattern is between the week 2 and week 1 gait patterns of MCLT + MMT rats and may represent a transition from unilateral to bilateral compensations (see below). By week 6, the gait patterns of both MCLT sham and MCLT + MMT animals were temporally symmetric, with some evidence of stance time imbalance and spatial asymmetry in MCLT + MMT animals.

Although the uninjured contralateral limb can compensate for an injured limb through unilateral compensations, bilateral gait compensations can also reduce the period of time during which a limb must bear weight without contralateral limb support [11]. Conceptually, this protective gait is similar to the way one might walk the day after a hard workout at the gym (when muscles are sore and tight in both limbs). Although this conceptualizes bilateral compensations as “the day after a hard workout,” these gait sequences are also protective. By “shuffle stepping,” stance times on both limbs are increased, effectively lengthening the periods for which both hind limbs are in ground contact (double-limb support) and reducing periods in which only one hind limb is in ground contact (single-limb support). As such, these gait sequences are often described by reduced periods of single-limb support and decreased stride lengths in both limbs of a limb system [11]. Although there was some evidence of unilateral compensations in our animals, there was strong evidence of bilateral gait compensations in both MCLT sham animals at 6 weeks postsurgery and MCLT + MMT animals at 4 and 6 weeks postsurgery, indicated by reduced stride lengths and reduced single-limb support phases on both hind limbs (*p* < 0.001 in all groups) (Fig. 5c).

To be clear, although bilateral compensations in rodents are often more difficult to identify than unilateral compensations, bilateral compensations should not be considered less significant in magnitude. Both unilateral and bilateral compensations protect an injured limb from loading by decreasing the period during which a limb must bear weight without contralateral limb support. This protection, whether through a unilateral or bilateral compensation, is easiest to observe through reduced periods of single-limb support. As an example, MCLT + MMT animals showed evidence of a unilateral compensation at week 1 by a reduced period of single-limb support on the injured limb only. At week 4 and week 6, both MCLT sham and MCLT + MMT animals showed evidence of bilateral compensation by reduced periods of single-limb support in both limbs (also see Fig. 2).

### Mechanical sensitivity

**Fig. 7:**
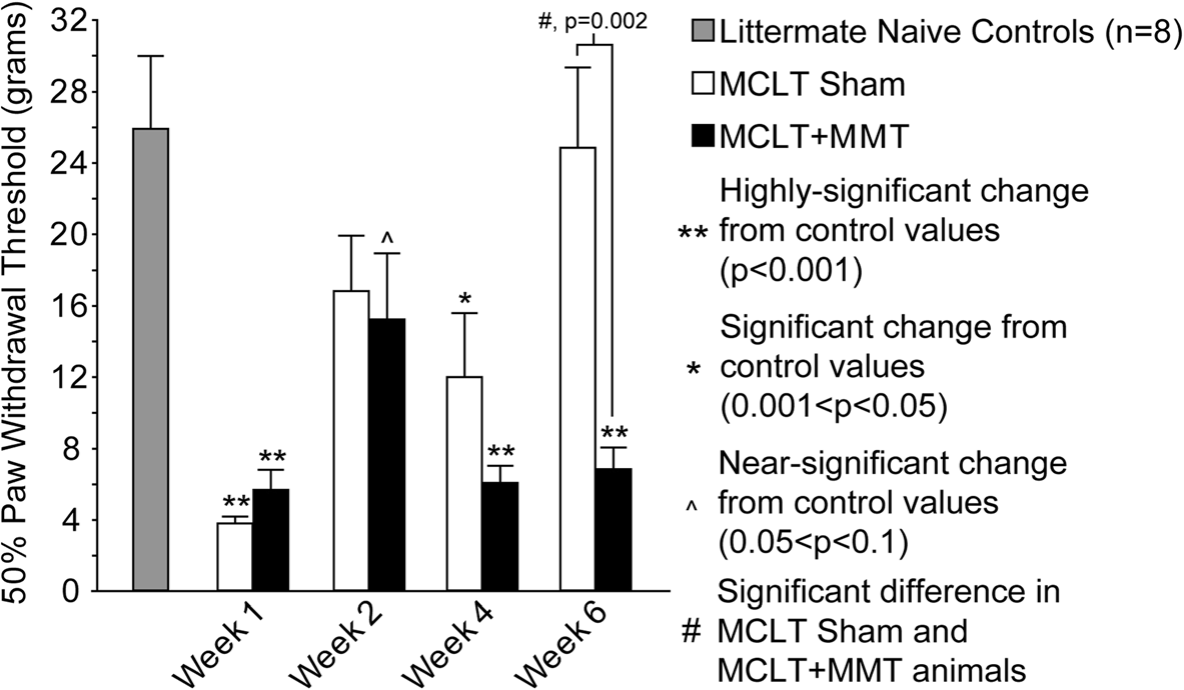
Tactile sensitivity changes following MCLT sham and MCLT + MMT surgeries in the rat. MCLT sham and MCLT + MMT surgeries cause heightened sensitivity in the operated limb at 1 week, indicated by a reduction in the paw withdrawal threshold relative to naïve control littermate levels (*p* < 0.001). Whereas animals with MCLT sham surgery recovered to control levels by week 6, animals with MCLT + MMT tended to maintain a heightened sensitivity across the time points, with significant reductions in the paw withdrawal threshold at week 4 and week 6 (*p* < 0.001 at all time points) and a tendency for heightened sensitivity at week 2 (*p* = 0.07). In addition, the paw withdrawal thresholds for animals with MCLT + MMT were significantly different from MCLT sham animals at week 6 (*p* = 0.002). Data are presented as mean ± SEM. *MCLT* medial collateral ligament transection, *MMT* medial meniscus transection

Animals in the MCLT group had heightened sensitivity to tactile stimuli at week 1 and week 4 (*p* < 0.001 and *p* = 0.009, respectively) (Fig. 7) but returned to near naïve control levels by week 6. Animals with MCLT + MMT had heightened sensitivity to tactile stimuli at weeks 1, 4, and 6 (*p* < 0.001 at all time points) and tended to have heightened sensitivity at week 2 (*p* = 0.07). In addition, the tactile sensitivity of animals with MCLT + MMT was significantly different from that of MCLT sham animals at week 6 (*p* = 0.002).

### Histology

**Fig. 8:**
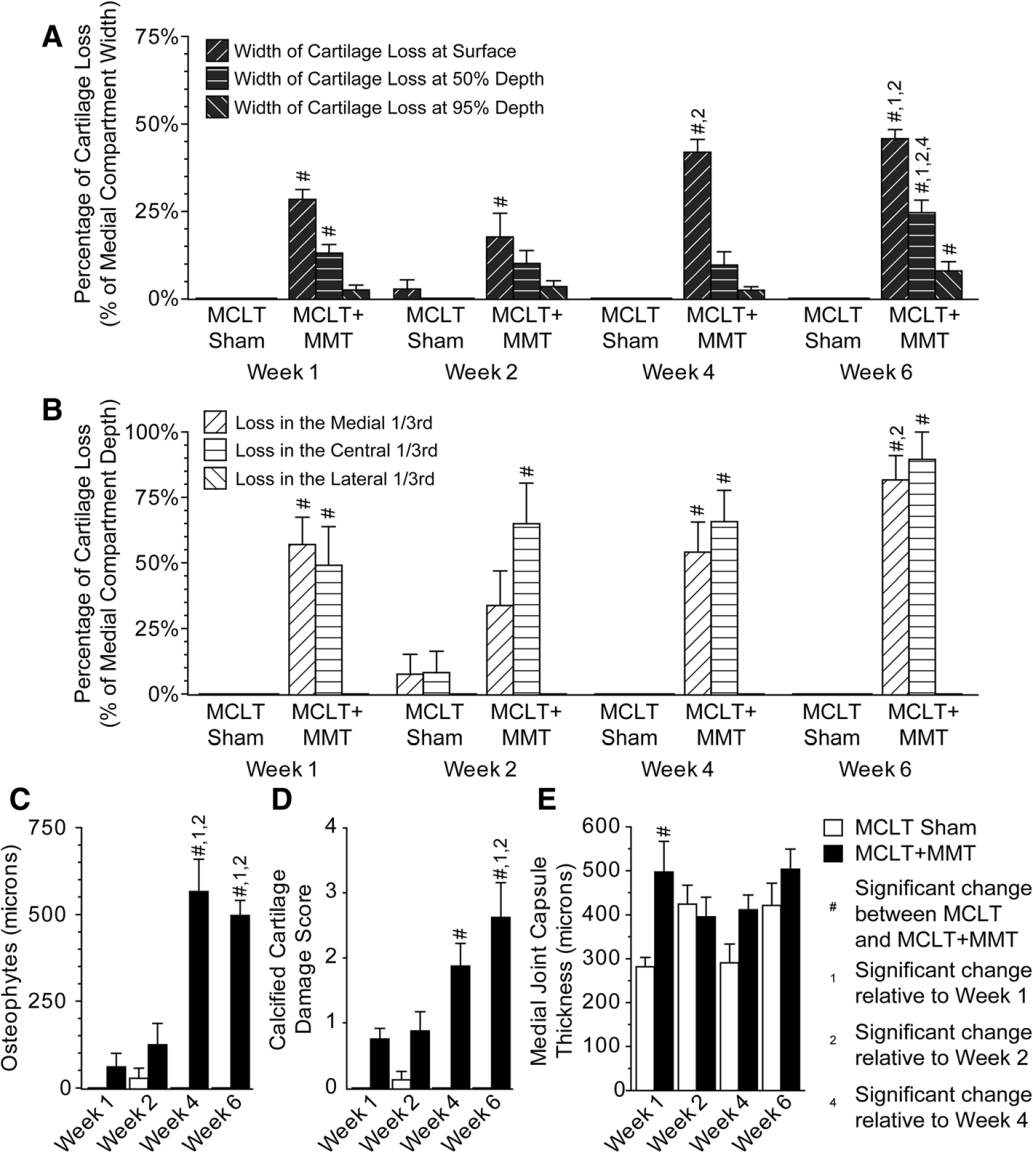
Cartilage damage and bone remodeling following MCLT and MCLT + MMT surgeries in the rat. Cartilage lesions were identified in the MCLT + MMT groups but not in the MCLT group. **a** The width of the cartilage lesion was significantly greater in the MCLT + MMT group than in the MCLT group at the cartilage surface at each time point (^#^ *p* < 0.04), and the width loss at 50 % depth and 95 % depth was wider than in the MCLT group at week 6 (^#^ *p* < 0.002). Moreover, the percentage of the surface affected increased over time in the MCLT + MMT group (^1,2,4^ *p* < 0.008). **b** The cartilage lesions were located primarily in the central and medial aspects of the medial compartment, with the depth of articular cartilage damage being significantly greater in the MCLT + MMT group than in the MCLT sham group in at least one location at all time points (^#^ *p* < 0.002). Moreover, the depth of the lesion progressed from nearly 50 % at week 1 to full thickness by week 6. **c** Significant growth of osteophytes was identified in the MCLT + MMT group at week 4 and week 6 relative to the MCLT control group (^#^ *p* < 0.001). Moreover, the size of the osteophytes was larger at week 4 and week 6 compared with week 1 and week 1 in the MCLT + MMT group (^1,2^ *p* < 0.001). **d** In addition, significant damage to the calcified cartilage was observed in the MCLT + MMT group relative to the MCLT group at week 4 and week 6 (^#^ *p* < 0.001), with the damage score in the MCLT + MMT group at week 6 being significantly higher than at week 1 and week 2 (^1,2^ *p* < 0.001). Differences in the medial capsule thickness between MCLT sham and MCLT + MMT animals were observed only at week 1 (^#^ *p* = 0.04, **e**). Data are presented as mean ± SEM. *MCLT* medial collateral ligament transection, *MMT* medial meniscus transection

**Fig. 9:**
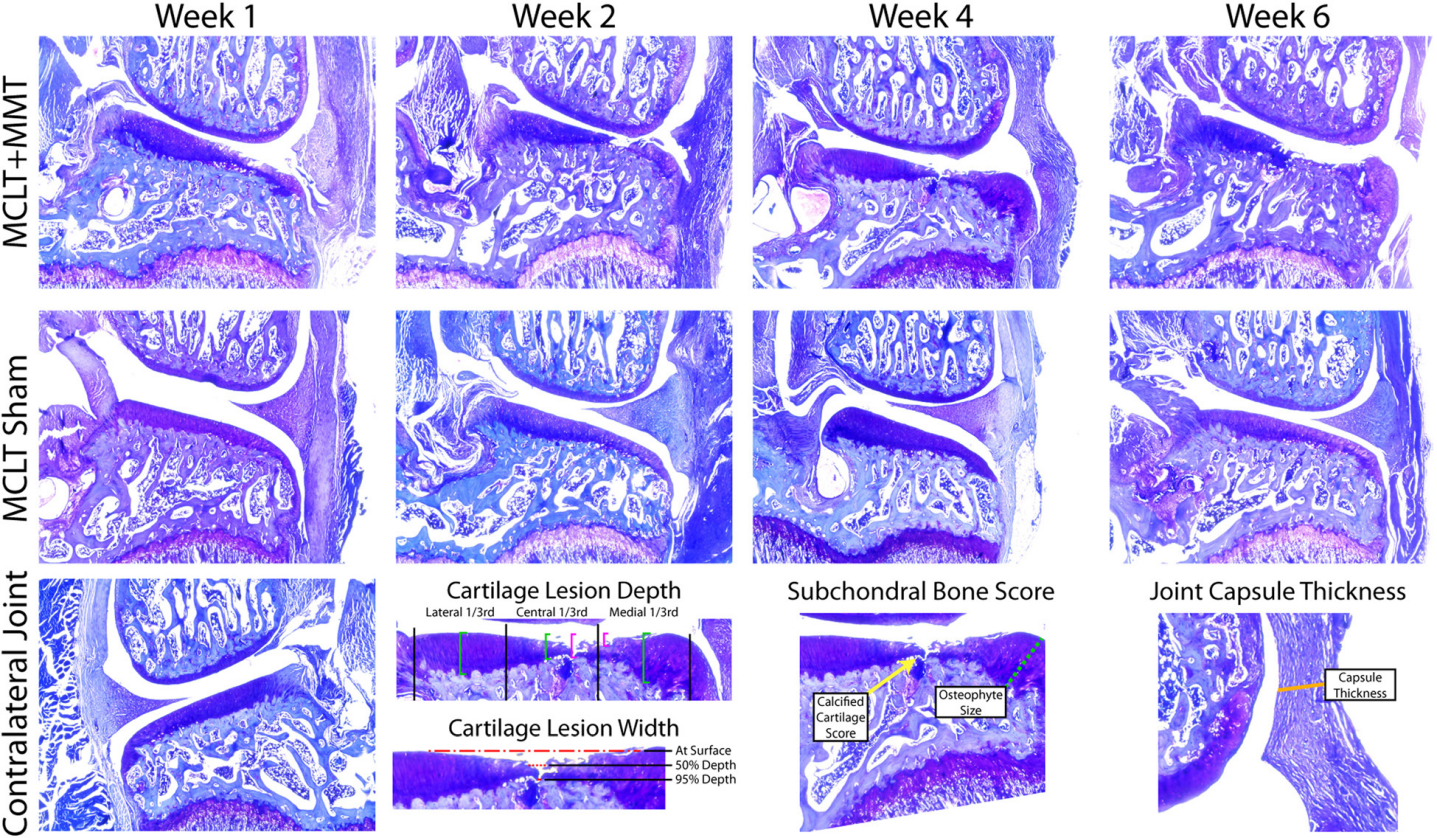
Representative histological images following MCLT and MCLT + MMT surgeries in the rat. Representative histological images are shown for each surgical group at each time point, with a representative image for a healthy control from the contralateral joint shown at *bottom left*. The cartilage lesion measurements described in Fig. 6 are visually described in the *bottom row*. Significant damage to the articular cartilage can be seen in the MCLT + MMT group, including cartilage loss, osteophyte growth, and damage to the calcified cartilage region. Very little cartilage damage or osteophyte growth is seen in the MCLT group. *MCLT* medial collateral ligament transection, *MMT* medial meniscus transection

Histological changes are graphically summarized in Fig. 8, and sample images from each time point are provided in Fig. 9. As expected, significant cartilage damage was observed in the MCLT + MMT group but not in the MCLT sham group. The percentage of the articular cartilage surface that showed evidence of fibrillation was significantly higher in the MCLT + MMT group relative to the MCLT sham at all time points (*p* < 0.04), with the severity of the lesion increasing over time (Fig. 8a). In general, the cartilage lesion was located in the central or medial aspects of the medial compartment, progressing from a maximum depth of 50 % at 1 week to nearly full-thickness lesions at 6 weeks (Fig. 8b). Coinciding with cartilage damage, significant osteophytes and calcified cartilage damage were found in the MCLT + MMT group at 4 and 6 weeks postsurgery (Fig. 8c, d). The MCLT + MMT group had a significantly thicker joint capsule at week 1 (*p* = 0.04).

### Univariate correlations

**Fig. 10:**
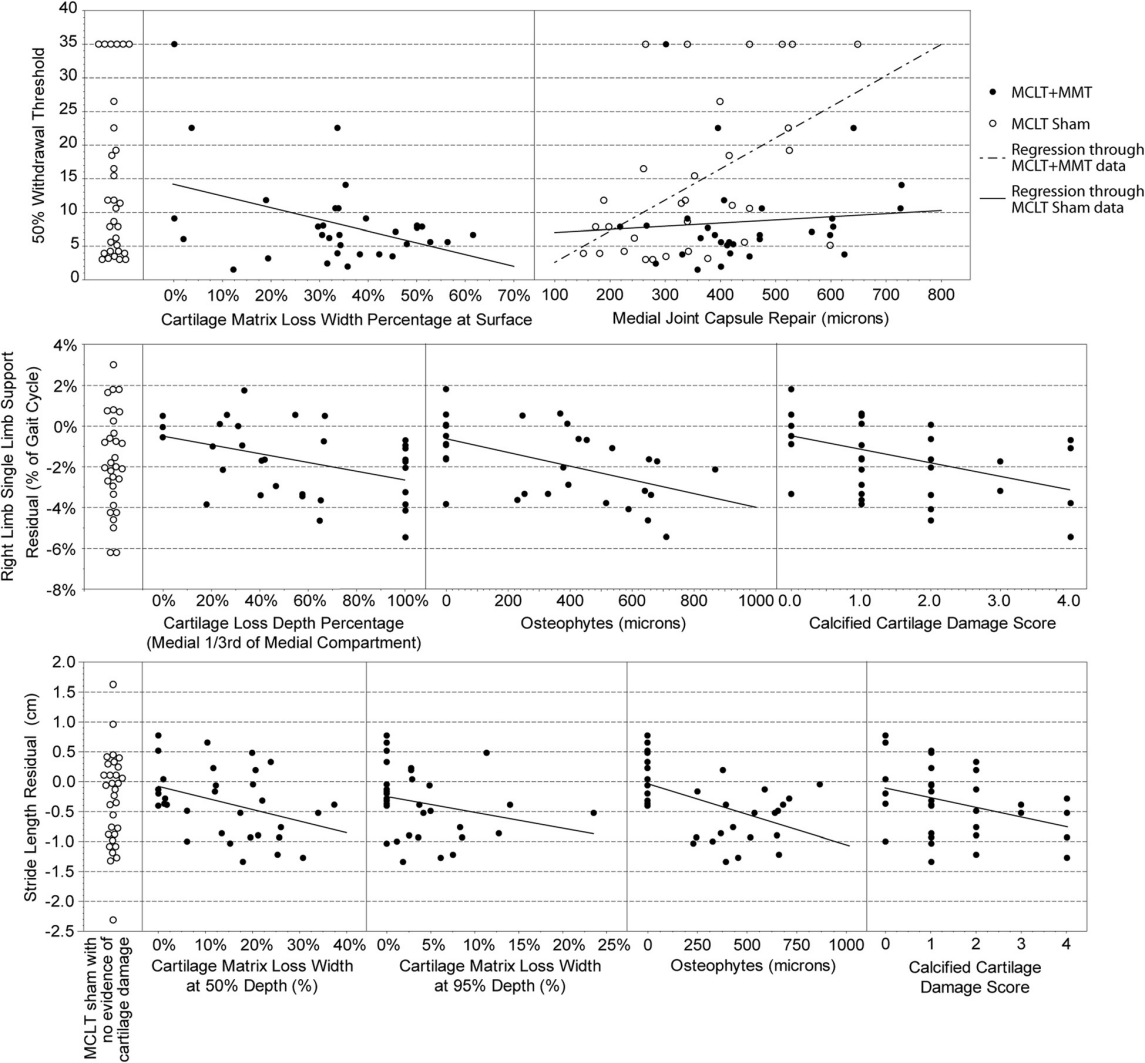
Visualization of correlations between histological evidence of joint damage and pain-related behaviors following MCLT and MCLT + MMT surgery in the rat. Whereas medial joint capsule repair correlated to tactile sensitivity in the MCLT sham group, medial joint capsule repair did not associate with tactile sensitivity in the MCLT + MMT group (*top*). Instead, cartilage matrix loss width at the surface correlated to tactile sensitivity in the MCLT + MMT group, despite evidence of cartilage damage in the MCLT sham group. Also, whereas correlations between stride length residual and injured limb (*right*) single-limb support phase could be identified in the MCLT + MMT group, similar gait changes could be identified in the MCLT sham despite evidence of joint damage (*middle* and *bottom panels*, respectively). *MCLT* medial collateral ligament transection, *MMT* medial meniscus transection

Correlations between histological changes in the joint and behavioral changes are shown in Additional File 1: Table S1, where the Pearson correlation coefficient *R* is shown on top and the *p* value for the slope term in the univariate model is shown on bottom. Owing to the lack of variance in histological measures, correlations could not be constructed for the MCLT sham group other than for joint capsule thickness; however, a positive correlation was seen between synovial capsule thickness and hind limb sensitivity in the MCLT sham animals (Fig. 10). This correlation was not repeated in the MCLT + MMT group; instead, a negative correlation was identified between the amount of the cartilage surface affected and the 50 % withdrawal threshold.

## Discussion

Although it is not life-threatening, OA is incurable and ultimately results in chronic, debilitating symptoms. Complicating clinical OA treatment is the common finding that the severity of joint degeneration does not necessarily correlate to the symptomatic consequences of OA [19–21]. Clinically, debilitating symptoms can appear across a broad spectrum of joint degeneration, where patients may experience intense pain or joint dysfunction with little or no radiographic evidence of tissue damage or minimal symptoms with severe radiographic evidence of tissue degeneration [22, 23]. The lack of a unifying relationship between cartilage damage and symptoms in patients with OA may be attributed to psychosocial conditions and individual coping capabilities [19, 24–26]. Stress and environmental factors can clearly affect OA pain experiences; however, a potential exists that cartilage loss simply does not explain a significant portion of OA symptoms, even if environmental factors are well controlled. Our data in this study provide an example of this conundrum in a rodent OA model, where environmental factors were well controlled between the OA and non-OA groups.

Among the correlations identified, many are counterintuitive and likely arbitrary associations. First, many researchers would postulate that a thicker synovial lining would occur as a result of synovial inflammation, and therefore a negative relationship should occur between joint damage and the 50 % paw withdrawal threshold (heightened sensitivity). We identified a positive correlation, not a negative one. Because the synovial capsule was damaged with MCLT + MMT surgery but not with MCLT sham surgery, we speculate that the thickening of the synovial lining may not occur until a later time in MCLT sham animals. Thus, the correlation identified in the MCLT sham group may be an arbitrary relationship that results from the recovery of limb sensitivity to baseline levels following surgery and the delayed development of synovial damage following MCLT sham surgery. However, a skin incision sham (which was not included in this study) or age-matched naïve controls would be necessary to verify this hypothesis. Similarly, stance time imbalance was negatively correlated to the cartilage lesion width and depth in MCLT + MMT animals. Because a limb imbalance greater than 0 indicates that more time is spent on the left limb (contralateral) than on the right limb (injured), most OA researchers would postulate that this correlation would be positive; namely, as the lesion size increases, the gait sequence becomes more imbalanced. Thus, like medial joint capsule thickness, correlations between limb imbalance and joint histology could be driven by arbitrary correlations resulting from the recovery of imbalance parameters to baseline levels following the MCLT + MMT surgery.

Negative correlations were observed between multiple histological measures and the stride length residual and single-limb support residuals. Conceptually, these correlations follow the anticipated relationship: As the joint damage increases in severity, stride lengths decrease and periods of single-limb support reduce. However, these same gait compensations were identified with MCLT sham surgery, despite a lack of damage identified through the OARSI histological grading system (Fig. 10). When MCLT sham and MCLT + MMT data sets were assessed in conjunction, the correlations in the MCLT + MMT group also appeared to be coincidental, despite following the predicted pattern.

To be clear, this experiment was specifically designed to compare animals receiving an MCL sham surgery with animals receiving an MCLT + MMT surgery. Rats began the experiment as littermates and were cohoused, tested side by side on the same day, and fed the same diet throughout the experiment. Whereas the gait profiles in these animals did differ from the expected gait pattern of naïve controls, minimal differences were observed between the OA cohort (MCLT + MMT) and the non-OA cohort (MCLT alone). Moreover, whereas correlations could be identified between joint damage and changes in animal behavior in the OA cohort (MCLT + MMT group), the same behavioral changes, by and large, were found in the non-OA cohort (MCLT sham), despite the lack of significant joint damage. The exception may be tactile allodynia, which remained elevated in MCLT + MMT animals at 6 weeks but was not elevated in MCLT sham animals. However, the correlations between hind limb sensitivity and cartilage damage in the OA cohort were relatively weak (*R* = −0.4498, *R*^2^ = 0.2023), and the range of sensitivities largely overlapped with the range in MCLT sham animals. Moreover, the tactile sensitivity difference between MMT + MCLT and MCLT sham animals did not appear until week 6, when cartilage damage was already very severe (>50 % of cartilage surface affected, cartilage lesions >75 % of the cartilage depth, and significant calcified cartilage damage). In combination, these data demonstrate the inherent limits of correlative relationships identified in our OA cohort. When taken in context with our MCLT sham results, correlations in the MCLT + MMT group appear to be largely coincidental or a relatively minor contributor to the behavioral phenotype in the OA group. Moreover, these data pose a fundamental question in the rat MCLT + MMT model of knee OA: Will therapeutics that prevent or reverse cartilage degeneration following a simulated meniscus injury have efficacy in treating OA-related symptoms and disability?

Although MCLT sham and MCLT + MMT animals were treated identically at each time point, data in a historical database of weight- and velocity-matched naïve animals were used as controls. Because of the strong correlations of most gait variables to animal weight and walking velocity (see Fig. 2), weight- and velocity-matched historical controls are advantageous relative to preoperative controls. Walking velocities can vary markedly between testing days and between trials, and most rats used in OA research gain 10–50 % body weight in the weeks after surgery. Failure to account for these covariates in the statistical analysis markedly reduces the sensitivity of the gait analysis [11]. The control database used in this study represents multiple experiments across a wide range of weights (307–425 g) and walking velocities (15.4–76.3 cm/s), allowing for the humane reduction of research animals by eliminating the need to replicate naïve data collected in prior work. Nonetheless, historical controls are not without limitations, as environmental factors can vary between the experimental animals and those in the control database. However, it is also worth noting that stride length and single-limb support time residuals consistently shifted down with postsurgical time, even though these data were collected from the cohorts in a random order (week 4, week 1, week 2, week 6). This indicates that the downward trend is unlikely to be due to a temporal change in the environment.

Unfortunately, the causes of gait abnormalities following MCLT sham and MCLT + MMT in the rat remain uncertain. Clearly, mechanical destabilization of the joint due to ligament injury of the MCL may cause a mechanical dysfunction that ultimately manifests in changes in the spatiotemporal gait pattern. However, if destabilization of the joint due to MCLT was the primary factor, gait compensations would be expected immediately after transection and for some dysfunction to occur consistently across the postsurgery time points. Instead, our data indicate a progressive development of bilateral gait compensations over time in both the MCLT sham and MCLT + MMT groups. This temporal shift seems to indicate that mechanisms other than mechanical loss of the MCL are involved in the development of the gait compensation found after these simulated joint injuries.

The act of cutting the skin, and not the injuries to the joint through either MCLT or MCLT + MMT, could cause the gait compensations and tactile sensitivity changes over the course of the 6-week experiment, and a skin incision sham would be needed to verify this hypothesis. Nonetheless, our primary conclusion is that, despite the development of full-thickness cartilage defects, calcified cartilage damage, and osteophyte formation in the MCLT + MMT group, there is no discernible gait pattern difference between the MCLT sham and MCLT + MMT groups, and differences in tactile sensitivity were limited to the 6-week time point. The lack of association between these histological parameters and rodent gait compensations would still hold even if the skin incision were the root cause of the gait abnormality, and this lack of association between cartilage damage and behavioral changes in a rodent model of OA highlights the lack of known unifying relationships between OA pathogenesis and the development of OA disease sequelae.

Another limit of this experiment includes the lack of preoperative data for von Frey testing; instead, data for naïve littermate controls were used. Although it would have been useful to understand how the tactile sensitivity of our animals changed over the course of the experiment, the primary intention of this experiment was to create a data set with behavioral profiles paired to a histological profile, such that correlations between behavior and histology could be constructed. In future studies, more sophisticated statistical correlation models may assist in identifying relationships between joint damage and behavioral changes, and assessment of changes in sensitivity, rather than raw sensitivity, may improve these correlations.

It is also worth noting that the von Frey test examines tactile sensitivity in the hind paw and that because the surgically simulated injury is at the knee, the von Frey test detects secondary (or referred) hypersensitivity. Knee bend or application of pressure to the knee could allow for more direct assessment of primary hypersensitivity in these models; however, it should be noted that these methods require animal restraint which may affect the behavioral measure.

For correlation analyses, the OARSI histopathology assessment scheme for the rat was advantageous because many of the parameters measured are real numbers rather than the ordinal ranks typical of many histological grading schemes. Nonetheless, the OARSI histopathology scheme still tends to be cartilage-centric and focuses largely on structural changes in the joint. This approach to grading joint damage may neglect the continuum of changes happening throughout the OA joint. First and foremost, proinflammatory and catabolic mediators are chronically upregulated in OA [6, 27, 28], and OA pain is often considered to be inflammatory. Inflammation also plays a critical role in MCL injury and repair after injury [29, 30], and upregulation of inflammatory mediators can affect muscle function. As such, behavioral changes associated with the MCL sham and MCLT + MMT surgery may be more closely linked to local inflammation at the site of each injury. Although joint inflammation was not directly assessed in this study, synovial capsule thickness (an indirect assessment of synovitis) did not appear to explain either gait or tactile sensitivity changes in the MCLT + MMT group. However, direct assessment of inflammatory cytokines and chemokines in the MCL, synovial fluid, synovial lining, or fat pad could be used in the future to more thoroughly evaluate the correlation between joint inflammation and behavioral changes.

In addition, strong evidence has emerged that OA pain has neuropathic pain components [31, 32], including evidence of damage to nociceptive fibers in the periphery of the joint [33, 34]. Joint innervation actively responds to the OA environment and joint injury [35, 36], and damaged sensory nerves can release neuropeptides that stimulate other nerve endings. Similarly, damage to the neuromuscular system can occur with MCL rupture [29, 37]. Because rats with MCLT sham surgery and MCLT + MMT surgery both experience chronic musculoskeletal injuries, behavioral changes observed in both models may be more indicative of nervous system remodeling, both in the periphery and centrally, rather than structural changes to the cartilage and bone within the joint. Again, direct assessment of changes in joint innervation and the associated dorsal root ganglia and dorsal horn of the spinal cord could be used in the future to more thoroughly evaluate the correlation between neuronal and behavioral changes in this model of OA.

Finally, movement-evoked pain is an early characteristic of OA, and, as a result, a person or animal may modify a gait pattern to protect an injured limb from loading and motion. If protective patterns are repeated over time, muscles surrounding the joint will adapt and a fear of specific movement patterns may develop. For chronic diseases such as knee OA, it is not yet clear if long-term use of a protective gait sequence promotes or prevents future joint degeneration; it is also not yet clear if the use of protective gaits or limb guarding will propagate OA-related disability. Thus, the gait abnormalities identified in this experiment are possibly learned behaviors that develop from the prior protection of an injured limb. In future experiments blocking joint afferents at the time of injury or after the onset of symptoms, researchers could begin to more thoroughly examine this hypothesis.

## Conclusions

The lack of unifying relationships between cartilage damage and symptoms in OA remains a significant obstacle to OA translation. The primary concern of patients with OA is pain and disability, and these disease sequelae cannot be concretely explained by joint structure. Although biopsychosocial models help explain the effects of stress and environmental factors on OA symptoms in patients, a potential exists that cartilage loss simply does not explain a significant portion of OA symptoms, even if environmental factors are well controlled. Our data in this study provide an example of this conundrum in a rodent OA model, where environmental factors are well controlled between the OA and non-OA groups. Whereas correlations could be identified between joint damage and changes in animal behavior in the OA cohort (MCLT + MMT group), the same behavioral changes, by and large, were found in the non-OA cohort (MCLT sham) despite the lack of significant joint damage. The exception may be tactile sensitivity, which remained elevated at 6 weeks in MCLT + MMT animals but not in the MCLT sham group. However, correlations between tactile sensitivity changes and cartilage damage were still relatively weak and did not appear until cartilage damage was already very severe. Although it is possible that different injuries result in similar disease sequelae in the rat, these data pose a fundamental question for preclinical OA models: Will therapeutics that prevent or reverse cartilage degeneration following a simulated joint injury have efficacy in treating OA-related symptoms and disability?

## Additional file

Additional file 1: Table S1. Correlations between histological evidence of joint damage and pain-related behaviors following MCLT and MCLT + MMT surgery in the rat: correlations between histological measures of joint degenerations (*left*) and behavioral measures of pain and disability (*top*) estimated using linear univariate models. The Pearson correlation coefficient (*R*) and the *p* value associated with the slope term (*p*) are shown for each tested correlation, with correlations with a *p* value less than 0.05 in *bold italics*. Several measures showed no variance; thus, the Pearson correlation coefficient cannot be calculated (represented by “--”). (JPEG 581 kb)

## Abbreviations

MCL: medical collateral ligament
MCLT: medial collateral ligament transection
MMT: medial meniscus transection
OA: osteoarthritis
OARSI: Osteoarthritis Research Society International
SLS: single limb support
