# Supplementary figures and images for: Spatiotemporal gait compensations following medial collateral ligament and medial meniscus injury in the rat: correlating gait patterns to joint damage
Source: Arthritis Res Ther. 2015 Oct 14;17:287. doi: 10.1186/s13075-015-0791-2 (PMC4604628; doi:10.1186/s13075-015-0791-2)

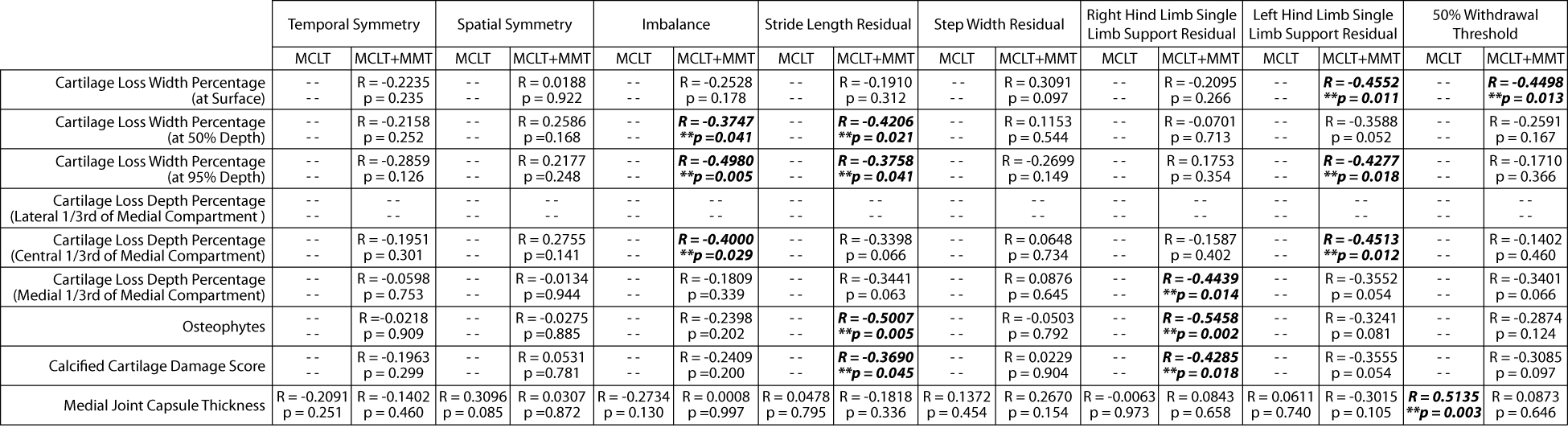

Supplement: Additional file 1: Table S1. — Correlations between histological evidence of joint damage and pain-related behaviors following MCLT and MCLT + MMT surgery in the rat: correlations between histological measures of joint degenerations (left) and behavioral measures of pain and disability (top) estimated using linear univariate models. The Pearson correlation coefficient (R) and the p value associated with the slope term (p) are shown for each tested correlation, with correlations with a p value less than 0.05 in bold italics. Several measures showed no variance; thus, the Pearson correlation coefficient cannot be calculated (represented by “--”). (JPEG 581 kb) [file 13075_2015_791_MOESM1_ESM.jpeg]
